# Immunological dysfunction of children with severe parapneumonic effusion

**DOI:** 10.3389/fped.2025.1547146

**Published:** 2025-06-27

**Authors:** Barnabás Rózsai, Diána Simon, Tímea Berki, Gabriella Kiss, Bernadett Mosdósi

**Affiliations:** ^1^ Department of Pediatrics, Medical School, University of Pécs, Pécs, Hungary; ^2^ Department of Immunology and Biotechnology, Medical School, University of Pécs, Pécs, Hungary

**Keywords:** parapneumonic effusion, community-acquired pneumonia, pneumococcal conjugate vaccine, immunodeficiency, children

## Abstract

**Purpose:**

Despite the worldwide decrease in the incidence of serious pneumococcal infections following the introduction of the 13-valent pneumococcal conjugate vaccines (PCV13), invasive infections still occur. This study aimed to investigate the immunological function of children with severe parapneumonic effusion (PPE) both during their hospitalization and after full recovery.

**Methods:**

This was a prospective, single-center study. Children with PPE were admitted to our clinic between 1 January 2011 and 30 June 2023, and participated in the study. Due to the severity of the effusion, all PPE cases required thoracic drainage and some children also underwent fibrinolysis and/or video-assisted thoracoscopic surgery. Demographic and clinical data and laboratory results were collected at admission. Extended immunological testing was performed at the time of clinical admission and again 6–8 weeks after discharge.

**Results:**

A total of 66 episodes of PPE were identified. During hospitalization, one patient was diagnosed with human immunodeficiency virus infection and another with immunoglobulin A deficiency. Extended immunological evaluation was performed during follow-up in 49 patients. Within this cohort, seven patients were diagnosed with mannose-binding lectin deficiency and three with specific antibody deficiency. In total, immune dysfunction was confirmed in 12 patients. When comparing the immunocompromised and non-immunocompromised groups, the duration of hospitalization was longer in the immunocompromised group, with no other differences observed.

**Conclusion:**

Although the incidence of severe PPE has declined since the introduction of PCV13, immunological evaluation remains essential for identifying underlying immunodeficiencies. Despite vaccination, screening patients with PPE for immune dysfunction is crucial. Early diagnosis and timely treatment can help prevent organ damage and reduce long-term morbidity.

## Introduction

Pleural effusions can stem from a variety of infectious and non-infectious disorders. Infection is the most common cause (50%–70%), with parapneumonic effusion (PPE) being a prominent example. Less frequent causes include malignancy and congestive heart failure (1).

Despite the widespread use of pneumococcal conjugate vaccines since the early 2010s, *Streptococcus pneumoniae* remains the leading cause of PPE worldwide (2). Asymptomatic nasopharyngeal carriage of this pathogen occurs in up to 60% of healthy young children (3), with even higher rates observed in low- and lower-middle-income countries (4). *S. pneumoniae* can cause a spectrum of illnesses, ranging from mild infections to severe diseases, such as pneumonia, PPE, septicemia, and meningitis, all associated with significant morbidity and mortality (5). PPE remains a severe complication of community-acquired pneumonia, with a considerable number of affected children requiring admission to the intensive care unit (2).

The introduction of the pneumococcal conjugate vaccine (PCV) between 2008 and 2010 significantly reduced both the incidence of pneumonia and its complications, including the PPE incidence rate (1). In the United States, the incidence of PPE peaked at 4.2 cases per 100,000 children annually prior to the PCV era. Following vaccine implementation, this number decreased to approximately two hospital admissions per 100,000 children per year between 2008 and 2014 (6). A similar trend was observed in the United Kingdom. Saxena et al. reported that, before the introduction of PCV13 in 2010, the annual hospitalization rate for PPE was 3.9 per 100,000 children. By 2013, this rate had declined to 1.9 per 100,000 children (7).

However, data also indicate that while the incidence of pneumococcal PPE has declined, there has been a corresponding increase in infections caused by *Streptococcus pyogenes* (8).

In patients with inborn errors of immunity (IEI), severe bacterial infections commonly manifest around 4 months of age, coinciding with the waning of maternally acquired immunoglobulins. IEIs represent a genetically and clinically heterogeneous group of disorders, currently comprising at least 500 genetically defined single-gene defects (9). The hallmark of immunodeficiency (ID) is increased susceptibility to infection; however, the affected organs and pathogens vary depending on the type of immune defect. In immunocompromised patients, invasive pneumococcal disease (IPD) can lead to life-threatening complications. A meta-analysis by van Aalst et al. found that the incidence of IPD is approximately 6–30 times higher in patients with different types of ID compared to healthy controls (10). Despite this, limited data are available regarding the immune function of children with PPE. The primary aim of this study was to evaluate the immunological function and immune response to infection in children with PPE. Additionally, we aimed to estimate the prevalence of different types of immune deficiencies within this patient population.

## Materials and methods

### Patients and methods

This was a single-center, prospective study conducted at the Department of Pediatrics, Medical School, University of Pécs. A total of 66 children (41 girls and 25 boys) were enrolled in the analysis between 1 January 2011 and 30 June 2023. Exclusion criteria included pleural fluid due to malignancy or trauma (hemothorax), iatrogenic causes of immunodeficiency (such as medications, nutritional status, or anatomic abnormalities), a known family history of immunodeficiency or autoimmune disease, and a history of previous bacterial infections.

The average age of the patients at admission was 4.8 ± 3.7 years (average ± SD). Before hospitalization, 60 patients (90.9%) had received prior vaccination with either PCV7 or PCV13, and 49 patients (74.2%) had received oral antibiotic treatment.

Upon hospitalization, all patients were treated empirically with parenteral antibiotic therapy with ceftriaxone (80 mg/kg/day), with or without clindamycin (40 mg/kg/day); this treatment was later adjusted based on microbiological results or the clinical response. Additionally, a thoracic drain was performed in all cases using an 8–12-Fr pigtail catheter. Indications for thoracic drainage included pleural fluid wider than 2 cm at the level of the hilus, the presence of septation or loculation in the pleural space (as detected by thoracic ultrasound), or severe respiratory distress in the presence of any pleural fluid. The thoracic drainage was initiated on the second (median) day of hospitalization [interquartile range (IQR): 1–3.5 days] and lasted for 6 (4–11.5) days. In cases of inadequate drainage or the appearance of loculation, intrapleural fibrinolytic therapy with tissue plasminogen activator (Alteplase, Genentech, USA) and/or thoracic surgery—either video-assisted thoracoscopic surgery (VATS) or decortication—was performed. Fibrinolytic treatment was administered in 56.1% (37/66) of cases. Due to insufficient conservative therapy, 18 patients (27.3%) required surgical intervention (decortication and/or VATS).

Extensive microbiological analysis of the pleural effusion was performed, utilizing standard culture, urinary antigen testing, and polymerase chain reaction (PCR) techniques. Simultaneously, blood culture was also obtained. At the beginning of hospitalization, an initial immunological screening test and routine blood tests, including complete blood count, C-reactive protein (CRP), procalcitonin, and blood culture, were performed. This included measuring serum immunoglobulin levels (IgA, IgE, IgG, IgM) and multiparameter flow cytometry analysis of lymphocyte subsets. Extended immunological evaluation was conducted 6–8 weeks after hospital discharge at the Allergy and Immunology Outpatient Care Unit of our clinic. At the outpatient unit, patients were clinically assessed, and peripheral venous blood samples were collected from all participants. Routine laboratory tests included complete blood count, CRP, serum immunoglobulin levels (IgA, IgE, IgG, IgM), and complement components (C3, C4). We also performed functional assessments of the classical, alternative, and lectin complement pathways. Additionally, peripheral blood lymphocytes were analyzed using multiparameter flow cytometry (Beckton Dickson and Company Biosciences, San Jose, USA). The following cell types were evaluated: CD56+ natural killer cells, CD3 + CD56+ natural killer T cells, CD3 + CD8+ cytotoxic and CD3 + CD4+ helper T lymphocytes, CD19 + CD5+ B1 and CD19 + CD5− B2 B lymphocytes, CD4 + CD25 high+ regulatory T and CD3 + CD25 medium+ activated T cells, CD3 + HLADR+ activated T cells, CD8 + HLADR+ activated T cytotoxic cell, CD3 + CD45RA+ naïve and CD3 + CD45RO+ memory T cells, CD19 + IgD + CD27− naïve B cells, and CD19 + IgD + CD27+ non-switched B and CD19 + CD27 + IgD− switched B cells. Specific antibody titers (against *S. pneumoniae*, tetanus, diphtheria, and *Haemophilus influenzae* type b) were also measured.

The classical complement pathway was assessed using a hemolytic complement assay with sheep red blood cells. The alternative and lectin complement pathways were evaluated by ELISA. The results for the lectin complement pathways were expressed as a percentage of a standard control, with values below 30% considered abnormal. Abdominal ultrasound was performed to exclude asplenia. Immunodeficiencies were defined according to the criteria of the International Union of Immunological Societies(11). Based on the results of the immunological investigations, the patients were categorized into two groups: those with and those without impaired immune function.

The study was approved by the Local Ethics Committee (Medical School, University of Pécs). Informed consent was obtained from the parents or legal guardians of all participants.

### Data analysis and statistics

SPSS for Windows 28.0 statistical software was used. Data distributions were tested for normality using the Kolmogorov–Smirnov test. Given that age, time and length of the drainage, lymphocyte count, the pleural fluid lactate dehydrogenase concentration, levels of the classical and alternative complement pathways, C3 and C4 levels, and pneumococcal antibody titer did not exhibit normal distribution, their median and IQR values are provided. For assessing statistically significant differences, Student's *t*-test (for normally distributed data), the Mann–Whitney test (for non-normally distributed data), and the *χ*^2^ test or Fisher’s exact test (for dichotomous variables) were used. A *p*-value of <0.05 was considered to be statistically significant.

## Results

### Clinical data

In this prospective study, *S. pneumoniae* was identified as the main etiological agent in 48 of 66 cases (72.7%). Only four patients (6.0%) required mechanical ventilation, while the remaining 60 children (90.9%) received supplemental oxygen therapy. The median duration of hospitalization was 16 days [interquartile range (IQR): 12–23.2], and no deaths occurred in the study population.

Seventeen patients were unavailable for follow-up and were therefore excluded from further analysis. Ultimately, 49 children were included in the extended immunological evaluation. There were no significant differences in age, sex, duration of drainage, length of oxygen therapy, or total hospital stay between those who participated in follow-up examinations and those who did not.

### Immunological data

Impaired immune function was identified in 12 of 49 patients (24.5%). Of these, seven patients were diagnosed with mannose-binding lectin (MBL) deficiency, three with specific antibody deficiency (SAD), one with IgA deficiency, and one with human immunodeficiency virus (HIV) infection. Demographic characteristics of patients with and without immunodeficiency are summarized in Table 1. No significant differences were observed between the two groups regarding age, gender, the start of drainage, or the frequency of use of fibrinolytic and VATS/decortication procedures. However, the duration of hospitalization was significantly longer in immunocompromised patients than in those without immune dysfunction [19.0 (16.5–22.5) vs. 14.0 (12–20) days; *p* = 0.006].

**Table 1:** Demographic data and clinical characteristics of the participants.

Variables	Patients with immune dysfunction	Patients without immune dysfunction	*p*
*n*	12 (24.5%)	37 (75.5%)	
Sex (male/female)	3/9	16/21	0.22
Age (years)	3.6 (3.1–5.0)	4.1 (3.1–4.9)	0.56
Prehospital antibiotic therapy	8 (66.7%)	29 (78.4%)	0.32
Start of drainage (days)	1.5 (1.0–3.0)	2.0 (1.0–3.5)	0.56
Length of drainage (days)	6.5 (5.0–15.5)	6.0 (4.0–9.5)	0.56
Length of hospitalization (days)	19.0 (16.5–22.5)	14.0 (12.0–20.0)	0.006
Fibrinolysis	6 (50%)	20 (54.1%)	0.81
Video-assisted thoracoscopy	4 (33.3%)	9 (24.3%)	0.39

Variables with not normal distribution are presented with median (IQR). For dichotomous variables, the *χ*^2^ test/Fisher’s exact test was used, and the Kruskal–Wallis test was used for non-normally distributed data.

Table 2 presents the pleural fluid characteristics and basic immunological findings of patients at ICU admission. Biochemical parameters of pleural fluid did not differ significantly between groups. Both groups exhibited elevated white blood cell and absolute neutrophil count levels without significant difference. Neutropenia was not observed in any patient. One 5-year-old patient, diagnosed with IgA deficiency, had an IgA level below 0.05 g/L. In the immunocompromised group, both IgM and IgG levels showed a non-significant increase. While median lymphocyte counts and subpopulations remained within the normal range, the percentage of CD3^+^CD25^+^ activated T cells was significantly higher in patients with immune dysfunction [2.8 (1.2–5.0) vs. 0.8 (0.7–1.8)%; *p* = 0.006]. Severe CD4^+^ lymphopenia (146/μl) was detected in a patient with confirmed HIV infection by serology.

**Table 2:** Common laboratory and basic immunological values at hospitalization of the participants.

Variables	Patients with immune dysfuntion	Patients without immune dysfunction	*p*
*n*	12 (24.5%)	37 (75.5%)	
C-reactive protein (mg/L)	261.6 ± 109.6	233.6 ± 100.7	0.21
Pleural fluid pH	6.98 ± 0.29	7.15 ± 0.25	0.055
Pleural fluid protein (g/L)	42.7 ± 4.7	40.7 ± 7.1	0.21
Pleural fluid LDH (U/L)	5,050 (1,220–19,300)	4,650 (910–10,970)	0.45
Immunoglobulin A (g/L)	2.36 ± 1.16	1.93 ± 0.88	0.11
Immunoglobulin M (g/L)	1.58 ± 0.54	1.29 ± 0.45	0.053
Immunoglobulin G (g/L)	14.4 (8.3–19.7)	9.6 (7.2–13.1)	0.33
White blood cells (/μl)	15 980 ± 6,550	19 720 ± 8,400	0.083
Absolute neutrophil count (/μl)	12 670 ± 5,660	15 870 ± 8,400	0.11
lymphocytes (/μl)	2,810 (1,560–3,200)	2,040 (1,310–3,320)	0.93
CD4+ T cells (/μl)	1,170 ± 630	1,210 ± 470	0.42
CD8+ T cells (/μl)	930 ± 400	800 ± 360	0.21
Active Th (%)	9.5 (4.8–24.8)	9.0 (6.7–13.1)	0.96
CD3/CD25+ T cells (%)	2.8 (1.2–5.0)	0.8 (0.7–1.8)	0.006
HLADR CD3+ T cells (%)	7.9 ± 8.5	5.9 ± 4.7	0.26
HLADR CD8+ T cells (%)	4.0 (1.5–11.4)	2.8 (1.8–5.2)	0.77
CD3/CD45RA+ T cells (/μl)	59.5 ± 12.6	56.2 ± 11.3	0.29
CD3/CD45RO T cells (/μl)	19.0 (12.9–34.5)	17.4 (14.5–22.2)	0.73
CD19+ lymphocytes (/μl)	373 (200–665)	400 (300–545)	0.48
Natural killer CD56+ cells (%)	3.9 (2.2–5.1)	4.2 (2.9–7.5)	0.48
Natural killer T cells (%)	2.5 (0.9–4.9)	1.4 (0.8–2.5)	0.29

Variables with normal distribution are presented as mean ± SD, and those with non-normal distribution are presented with median (IQR).

Table 3 details the extended immunological findings from outpatient follow-up. The total CD19^+^ naïve B lymphocyte counts and the age-matched Ig levels were within the normal range and did not differ significantly between groups. However, three patients exhibited low pneumococcal antibody concentrations (19.6, 26.3, and 37.2 mg/L; normal range: >50 mg/L) despite confirmed severe infection and vaccination. Since revaccination did not increase antibody titers, they were diagnosed with specific antibody deficiency. All three patients began immunoglobulin replacement therapy.

**Table 3:** Detailed immunological characteristics of patients at the outpatient unit.

Variables	Patients with immune dysfunction	Patients without immune dysfunction	*p*
*n*	12 (24.5%)	37 (75.5%)	
Immunoglobulin A (g/L)	1.15 (0.62–1.78)	1.04 (0.7–1.45)	0.33
Immunoglobulin M (g/L)	1.16 (0.9–1.8)	1.1 (0.8–1.42)	0.42
Immunoglobulin G (g/L)	9.9 ± 3.7	9.8 ± 2.3	0.46
Pneumococcal antibody level (mg/L)	62.7 (26–313)	107 (70.2–323.4)	0.28
CD3+ lymphocytes (/μl)	2,900 ± 1,490	2,730 ± 1,090	0.35
CD4+ T cells (/μl)	1,470 ± 820	1,330 ± 560	0.30
CD8+ T cells (/μl)	1,030 (578–1,478)	1,060 (817–1,445)	0.89
Active Th (%)	16.8 ± 8.8	13.7 ± 8.5	0.16
CD3/CD25+ T cells (%)	1.9 (0.75–3.5)	1.6 (0.9–3.2)	0.59
HLADR CD3+ T cells (%)	4.4 (2.9–8.2)	5.6 (4.0–9.2)	0.38
HLADR CD8+ T cells (%)	4.4 (2.5–6.3)	4.1 (2.4–7.2)	0.59
CD3/CD45RA+ T cells (/μl)	48.2 ± 16.3	46.9 ± 8.8	0.41
CD3/CD45RO T cells (/μl)	26.0 ± 11.5	28.3 ± 8.3	0.24
CD19+ lymphocytes (/μl)	284 (164–518)	332 (217–403)	0.52
Natural killer CD56+ cells (%)	12.6 ± 8.8	10.0 ± 5.5	0.20
Natural killer T cells (%)	1.3 (0.6–2.5)	1.9 (1.1–4.8)	0.33
Complement 3 (g/L)	1.19 ± 0.26	1.2 ± 0.24	0.40
Complement 4 (g/L)	0.24 (0.18–0.35)	0.22 (0.18–0.32)	0.46
Classical complement pathway (%)	61.2 ± 21.6	55.5 ± 17.7	0.19
Alternative complement pathway (%)	80.5 (71–92)	82 (59–92)	0.45
Lectin pathway (%)	44.4 ± 49.0	78.8 ± 41.6	0.013

Results of detailed immunological analysis among patients with or without immunodeficiency after 6–8 weeks of hospitalization. Variables with normal distribution are presented as mean ± SD, and those with non-normal distribution are presented with median (IQR).

Antibody titers for tetanus, diphtheria, and *Haemophilus* were within the normal range in both groups. T-cell numbers, subsets, and activation markers were also within the normal range, with no significant difference observed between groups.

The classical and alternative complement pathways were within normal ranges; however, seven children exhibited reduced lectin pathway activity and were diagnosed with MBL deficiency.

No cases of asplenia were detected by abdominal ultrasound examination.

## Discussion

To our knowledge, the study represents the first investigation into extended immune function within a homogeneous cohort of children presenting with severe PPE and requiring intensive care treatment. Our screening protocol revealed that approximately one in four children with severe PPE exhibited some degree of immune dysfunction.

Severe infections can occur in vulnerable patients who are immunocompromised by immunosuppressive treatment, HIV infection, or chronic inflammatory diseases (12). For cases where no identifiable risk factor is apparent at presentation, comprehensive immunological exploration is crucial for detecting potential primary or acquired immunodeficiencies (13). While severe bacterial infection may be the initial presenting symptom of IEI, immunological screening is not yet standard practice in many clinical settings (14).

A retrospective study by Strasser et al. identified significantly impaired immune function in 6% of pediatric patients with severe bacterial infection requiring intensive care (15). Furthermore, an additional 11% of subjects exhibited mild immunological abnormalities, which were potentially attributable to delayed immune system maturation. This suggests that approximately 17% of their heterogeneous patient population—which included children with sepsis, meningitis, and PPE—demonstrated varying degrees of immune dysfunction. Our prospective study, focusing exclusively on a homogeneous PPE cohort, found a similar prevalence (15). Previous studies indicate that 1%–10% of children presenting with invasive pneumococcal disease possess an underlying IEI (16–18).

Pulmonary disease is common among patients with IEI (19). While individual disorders are rare, collectively, they represent a significant cause of morbidity and mortality (20).

*S. pneumoniae* is the primary pathogen responsible for PPE. The severity and localization of pneumococcal infection vary from asymptomatic colonization to invasive disease. The pathomechanism underlying the progression from asymptomatic colonization to invasive pneumococcal infection remains poorly understood. Factors such as strain-specific virulence and the presence of underlying chronic disorders or immunodeficiencies may contribute to this progression (21).

The immune defense against pneumococcal infection involves complex pathomechanisms. The innate immune system functions as the first line of defense. Peptidoglycans found in Gram-positive bacteria primarily activate the complement system via the alternative pathway. Additionally, bacteria expressing mannose residues on their surface can bind MBL, thereby facilitating opsonization and activating the complement system via the lectin pathway (22). MBL, a soluble pattern recognition molecule, contributes to the elimination of a broad spectrum of pathogenic microorganisms by activating the lectin complement pathway and promoting opsonophagocytosis (22).

The synthesis of MBL is influenced by polymorphisms in the *MBL2* gene. However, serum MBL levels are not solely determined by genotype, as other genetic and environmental factors also affect their expression. Therefore, measuring serum MBL levels is considered a more reliable method for diagnosing MBL deficiency than relying solely on genotyping (23). Clinical studies have demonstrated that MBL deficiency predisposes individuals to severe respiratory tract infections (24).

Nasopharyngeal carriage of *S. pneumoniae* is approximately twice as prevalent in MBL-deficient children compared to healthy controls (25). Furthermore, MBL deficiency plays a more prominent role in infection susceptibility in individuals with predisposing risk factors, such as chronic lung disease and cystic fibrosis, and in infants with immature adaptive immune systems (24). The absence of these above-mentioned risk factors in our patients underscores the importance of comprehensive immunological screening.

Several studies have reported an increased risk of invasive pneumococcal disease in MBL-deficient children and adults (26–28). In our study, MBL deficiency was the most common immunodeficiency identified. Although no serious infections occurred during the follow-up period, upper respiratory tract infections were more frequent in these patients than in healthy controls. Currently, no specific therapy exists for this complement deficiency; however, immunization of family members and household contacts is recommended to reduce the risk of infections (29).

The cellular immune response also plays a critical role in protection against pneumococcal infection (30). The Th17 response has been shown to be a key component of pneumococcal immunity in both mouse models and immunodeficiency conditions, such as hyper-IgE syndrome (31, 32). Serum immunoglobulin E levels in our patients were below 2,000 IU/ml, thereby effectively ruling out hyper-IgE syndrome. The severe CD4^+^ lymphopenia in our HIV-infected patient further highlights the importance of screening for secondary immunodeficiencies. The significantly elevated CD3^+^CD25^+^ T-cell count within the immunocompromised group is likely indicative of immunocompensatory mechanisms.

Recurrent sinopulmonary infections represent a typical symptom of humoral immune deficiency (33), which is the most common form of IEI. This condition can result from defects in B-cell development, abnormal B-cell activation, or impaired antibody synthesis. Screening for humoral IEI typically involves flow cytometric analysis of B-cell subsets, quantitative measurement of serum immunoglobulin levels, and evaluation of specific antibody responses to infection and/or vaccination (34, 35).

Within our cohort, one patient was diagnosed with selective IgA deficiency (sIgAD). Most individuals with sIgAD are asymptomatic or experience mild sinopulmonary and gastrointestinal infections (36). However, Gaschignard et al. reported a notably higher prevalence of IgA deficiency (5%–7%) in children with severe bacterial infection compared to the general population (<1%) (16). It is important to emphasize that during immunological follow-up, 20%–30% of patients with IgA deficiency may develop autoimmune disorders, such as celiac disease or thyroiditis (37).

Three patients were diagnosed with SAD, which is characterized by an isolated, impaired antibody response to polysaccharide antigens despite normal levels of serum IgG, IgG subclass, IgA, and IgM, as well as normal T-cell subpopulations (38). Several monogenic disorders, including common variable immunodeficiency disorders, ataxia telangiectasia, Wiskott–Aldrich syndrome, and nuclear factor-kappa-B essential modulator deficiency, are associated with SAD. Identification of these patients is critical for appropriate management and prevention of complications. Early antibiotic treatment and regular immunoglobulin replacement therapy can prevent long-term complications such as mastoiditis and bronchiectasis (39). In our cohort, we observed no infections after initiating immunoglobulin substitution therapy. In addition to infectious complications, non-infectious manifestations such as autoimmunity and hematopoietic malignancies have also been reported in patients with SAD.

Rare primary immunodeficiency syndromes may also predispose patients to severe pneumococcal infections. Autosomal recessive disorders, such as NF-kappa-B essential modulator (NEMO) or NF-kappa-B alpha inhibitor (IKBA) deficiency, along with IL-1 receptor-associated kinase type 4 (IRAK-4) and myeloid differentiation primary response 88 (MyD88) deficiency, disrupt Toll-like receptor and interleukin-1 receptor signaling pathways, leading to increased susceptibility to severe infections (40).

In our study, it was not possible to investigate interleukin 6 production by white blood cells; therefore, the presence of MyD88 deficiency and disruptions in Toll-like receptor signaling were not assessed. Additionally, genetic testing was not performed on our patients. It is worth noting that, however, genetic testing is not routinely performed in clinical practice (41).

Primary immunodeficiency diseases and acquired immunodeficiencies can also predispose to severe infection. HIV infection is the most common acquired immunodeficiency. The prevalence of pleural effusion among patients hospitalized with AIDS ranges from 2% to 20%, often caused by bacterial infection, tuberculous pleuritis, or other malignancies (42). *Staphylococcus aureus* was more common in the HIV-positive group, along with anaerobic and opportunistic infections. In our study, the patient's severe infection, dystrophic appearance, and severe stomatitis raised suspicion of HIV infection, which was subsequently confirmed by serological testing.

## Conclusion

Pulmonary disease is a common manifestation of many IEIs and continues to be a leading cause of morbidity and mortality. In our study, varying degrees of immune impairment were identified in approximately one-quarter of children presenting with severe PPE. We advocate for detailed immunological screening in children with severe PPE. Early diagnosis and timely initiation of appropriate treatment are crucial for improving the prognosis of patients with primary immunodeficiencies.

## Study limitations

Our study has several limitations. First, it single-hospital setting may limit the generalizability of our findings. Second, not all children with PPE were tested for detailed immune function; therefore, the true prevalence of immunodeficiencies in this population may differ from that reported here. However, basic physiological and laboratory parameters did not significantly differ between the tested and untested groups. Additionally, pneumococcal serotyping of patients has only been feasible in recent years, so serotype data are not available for all cases. Among the serotyped isolates, serotype 3 was the predominant strain (unpublished data).

## Data Availability

The raw data supporting the conclusions of this article will be made available by the authors without undue reservation.
